# Numerical simulation studies of the new quaternary MAX phase as future engineering applications: The case study of the Nb_2_ScAC_2_ (A = Al, Si) compounds

**DOI:** 10.1038/s41598-023-49172-4

**Published:** 2023-12-22

**Authors:** Ahmed Azzouz-Rached, Mohammed Bendjemai, Mudasser Husain, Ali Bentouaf, Hamza Rekab-Djabri, Vineet Tirth, Ali Algahtani, Tawfiq Al-Mughanam, Abdulaziz H. Alghtani, Hussein Alrobei, Muawya Elhadi, Nasir Rahman

**Affiliations:** 1 Magnetic Materials Laboratory, Faculty of Exact Sciences, Djillali Liabes University of Sidi Bel-Abbes, Sidi Bel-Abbes, Algeria; 2 Physical Engineering Laboratory, University of Tiaret, 14000 Tiaret, Algeria; 3 Department of Physics, University of Lakki Marwat, Lakki Marwat, Khyber Pukhtunkhwa, 28420 Pakistan; 4 Faculty of Technology, Dr. Moulay, Tahar University of Saida, 20000 Saida, Algeria; 5 Faculty of Nature and Life Sciences and Earth Sciences, AkliMohand-Oulhadj University, 10000 Bouira, Algeria; 6 Mechanical Engineering Department, College of Engineering, King Khalid University, 61421 Abha, Asir, Kingdom of Saudi Arabia; 7 Research Center for Advanced Materials Science (RCAMS), King Khalid University, Guraiger, 61413 Abha, Asir Kingdom of Saudi Arabia; 8 Department of Mechanical Engineering, College of Engineering, King Faisal University, P. O. Box 380, Al-Ahsa, 31982 Kingdom of Saudi Arabia; 9 Department of Mechanical Engineering, College of Engineering, Taif University, P.O. Box 11099, Taif, 21944 Kingdom of Saudi Arabia; 10 Department of Mechanical Engineering, College of Engineering, Prince Sattam Bin Abdul Aziz University, Al-Kharj, 11942 Kingdom of Saudi Arabia; 11 Department of Physics, Faculty of Science and Humanities, Shaqra University, P. O. Box 1040, Ad-Dawadimi, 11911 Kingdom of Saudi Arabia

**Keywords:** Materials science, Physics

## Abstract

Recently, MAX phases have attained considerable technological interest owing to their two inherent properties metallic and ceramic properties. This study extensively examined Nb_2_ScAC_2_ MAX phases using DFT, to assess the structural, mechanical, electronic, and Thermal characteristics. Firstly, the stability of these two compounds was confirmed through the formation energy, elastic constants (C_ij_), and phonon band structure, which confirmed their thermodynamic, mechanical, and dynamical stability. The optimized lattice parameters of these compounds were examined and then utilized to calculate the physical properties of the Nb_2_ScAC_2_ compound. Our compounds are brittle due to their Pugh’s ratio of less than 1.75. The covalent bonding of the structure revealed by the Poisson ratio is less than 0.25 for the two compounds. The Nb_2_ScAC_2_ material is anisotropic, and Nb_2_ScAlC_2_ is harder than Nb_2_ScSiC_2_.The metallic character of the materials was affirmed by the electronic band structure analysis. Calculated thermal properties such as Debye temperature and minimum and lattice thermal conductivity reveal that both compounds have the potential to enhance their deployment in thermal barrier coating materials. On the other hand, the high melting temperatures indicate that our compounds could potentially be utilized in demanding or severe conditions. Finally, the thermodynamic characteristics, comprising the isochoric heat capacity (C_v_) and Debye temperature (ϴ_D_) were analyzed subjected to high temperatures and pressures. The optical constants such as real and imaginary parts of the dielectric function, refractive index and reflectivity, are investigated. The current study recognizes these two compounds as promising candidates for utilization in modern technologies and diverse industries.

## Introduction

Recently, the MAX phase has garnered significant interest in the cutting-edge world due to its excellent mechanical and thermal properties of these compounds at elevated temperatures, exhibiting characteristics of both metals and ceramics^1–3^. Consequently, it has found extensive applications in diverse industrial fields, including machinable refractories, ductile heating elements operating at elevated temperatures, coatings for electrical contacts, and nuclear-resistant components for neutron irradiation. Recently, certain materials have exhibited potential as Thermal Barrier Coatings (TBCs)^4–7^. The MAX phases are an ideal option for high-temperature TBC applications^8^, Ta_2_AlC and Cr_2_AlC have been identified significant potential for use as thermal barrier coatings due to their low minimum thermal conductivity, which can be at a minimum value of 0.855 W/(m–K) and 0.963 W/(m–K) respectively. In addition, Ta_4_AlC_3_ has a lattice thermal conductivity of 5 W/m–K and an experimental value of 6 W/m–K at 1300 K, while Nb_4_AlC_3_ has a lattice thermal conductivity of 7 W/m–K at 1300 K^9^. Efforts to develop improved TBC materials are hindered by the inadequate information on the thermal and mechanical properties of MAX phases. Comprehending the transfer of properties within the MAX phase oxide family and establishing an effective screening method are essential to accelerate the creation and identification of TBC materials based on MAX phases^10–12^. Materials containing Niobium (Nb) are attracting attention due to their demonstrated usefulness in various modern technologies, diverse industries, and structural transitions. They also enable the application of exceptionally high-temperature Thermal Barrier Coatings (TBC)^13^. One notable example is the incorporation of Nb on the M-site, which provides an opportunity to synthesize metallic-based nano-lamellar materials with desirable electronic properties^14^. A study by M.A. Ali et al*.* the physical properties of the new MAX phase borides Nb_2_SB were studied, and they showcased the best combination of mechanical properties in bulk crystal form^15^. In the current year, P. Das et al. published a report on the physical properties of the Nb_2_AC (A = Ga, Ge, Tl, Zn, P, In, Cd, and Al) 211-MAX phase compound. Their findings indicated that this compound has potential suitability for applications as solar radiation-protecting coatings and thermal barrier coating (TBC) materials^16^. Furthermore, M. R. Rana et al. discovered that MAX phase borides, specifically M_2_GaB (M = Sc, V, Nb, Ta) compounds, have the potential to serve as coating materials for the purpose of reducing solar heating^17^. In the aforementioned applications of MAX phases, several specific materials have been studied. Some of the notable materials investigated include: (Ti_1-x_Mox)_2_AlC (0 ≤ x ≤ 0.20)^18^, Ti-based M_2_AX phases 314-MAX phase boride Zr_3_CdB_4_^19^, 27 S-based M_2_SX (M = Ti, Zr, Hf, V, Nb, Ta, Cr, Mo, W; X = B, C, N)^20^, Te-containing layered ternary Hf_2_TeB MAX phase^21^. MAX phases are a large family of materials; they are more than one hundred and fifty various compositions have been studied and researched, over the past 25 years^22–26^. They are structured in layers and present a unique combination of properties. Despite their excellent properties, their industrial utilization is limited by the main factors: (i) it takes a significant amount of time to obtain licenses for products in industries. (ii) Large and complex family of materials. (iii) Higher pure commercial powders are unavailable^27^. Actually, MAX phases are structured in atomic layers forming both metallic and covalent bonding responsible of their ceramic and metallic character. Their structure consists of « M_n+1_X_n_ » ceramic layers where n = 1, 2, and 3 intercalated layers of metallic "A-group elements" that are separated by layers that are only about one atom thick. The M-X bonds in MAX phases are robust, whereas the M-A bonds are comparatively weaker. All MAX phases exhibit a hexagonal crystal structure characterized by the space group P6_3_/mmc (N° 194)^28^. The MAX phases are distinguished by a close-packed arrangement of “M” atoms interpolated with A-group atomic laminae, with “X” atoms occupying the octahedral positions between the M layers. Because of this arrangement, the MAX phases possess laminated layered structure, which qualifies them as nano-laminates. The researchers have suggested that the chemical, physical, and mechanical properties of MAX phase M_n+1_AX_n_ can be improved by introducing double transition metal. As a result, MAX phase compounds hold the potential for enhancing the characteristics exhibited by this category of materials for future engineering applications by adding double transition metal on “M” sites^29^. MAX phase compounds have recently gained significant attention from the scientific community due to the potential to customize their physical properties by adding a fourth element to the M-site. As a result, the expansion of their research has resulted in the establishment of a new research wing and the broadening of their field of applications. The compounds have demonstrated significant improvements in essential properties including oxidation resistance, fracture toughness, strength, and self-healing capabilities. Furthermore, both theoretical and experimental efforts have been undertaken to enhance the hardness and strength of quaternary MAX phases. For instance, new products such as Cr_2_TiAlC_2_^30,31^, Mo_2_ScAlC_2_ and Mo_2_TiAlC_2_^32,33^ have been synthesized. A recent study by Ouadha et al*.* presented a comprehensive theoretical analysis of a new ferromagnetic quaternary Mn_2_VSnC_2_ compound, which demonstrated both mechanical and thermodynamic stability in the α-polymorph ferromagnetic arrangement. The study also revealed that the compound has potential applications in harsh environmental conditions^34,35^. Benjemai et al. pursued a similar approach in their study of V_2_ScSnC_2_ and Nb_2_ScSnC_2_ compounds, examining several of their physical properties and proposing potential applications in modern contexts, such as high-energy applications^36^. Therefore, conducting theoretical research on other materials is of great significance. The present study aims to explore the structural, electronic, mechanical, lattice dynamic and optical properties and verify the stability of V_2_ScSnC_2_ and Nb_2_ScSnC_2_ compounds for the first time. Based on these properties it can be decided whether Nb_2_ScAC_2_ be a suitable candidate material for high energy applications. The following sections are arranged as: Section "Details of computational methodology" describes the method of calculations; the results are described with implications in Section "Results and discussions". Finally, in Section "Conclusions", concluding remarks are presented.

## Details of computational methodology

The FP-LAPW scheme has been utilized all over this study^37–39^, coded within WIEN2K^40^. We adopted the (GGA-PBE) for the exchange–correlation functional^41^. Therefore, the input parameters must be chosen in the sake of obtaining good precisions of the results. An R × K_max_ value of 8 has been utilized. Similarly, the maximum values of l_max_ = 10 and G_max_ = 14 have been chosen for the largest G-vector in charge density Fourier expansion and for the magnitude of “l” for the partial waves within atomic spheres, respectively. For the R_MT_ radii for Niobium (Nb), Scandium (Sc), Aluminium (Al), Silicon (Si) and carbon (C) we have chosen 1.97, 2.2, 2.5, 2.2 and 1.62 respectively. The SCF-cycles are performed iteratively until convergence energy reached 10^–5^ Ry and the same for charge convergence. We have used the Monkhorst–pack method to sample the irreducible Brillouin zone of our predicted compounds, with 9 × 9x2 special k-points. The cut-off energy of -6 Ry was selected to establish the separation of valence and core states. The thermodynamic properties were computed using the quasi-harmonic Debye model approximation, which was executed in the Gibbs2 software^42^.

The elastic parameters were determined using the measured elastic constants and the Voigt-Reuss-Hill approximations^43^. The following relations define these elastic parameters:

1 $$B_{V} = \left( {C_{11} + C_{12} } \right) + 2\left( {C_{13} + C_{33} } \right)$$

2 $$G_{V} = \frac{{\left( {C_{11} + C_{12} - 4C_{13} + 2C_{33} + 12C_{44} + C_{66} } \right) }}{30}$$

3 $$B_{R} = \frac{{\left( {C_{11} + C_{12} } \right)C_{33} - 2C_{13}^{2} }}{{C_{11} + C_{12} + 2C_{33} - 4C_{13} }}$$

4 $$G_{R} = \frac{5}{20} \times \frac{{C_{55} C_{66} \left[ {\left( {C_{11} + C_{12} } \right)C_{33} - 2C_{13}^{2} } \right]}}{{3B_{V} C_{44} C_{66} + \left\{ {\left\{ {\left( {C_{11} + C_{12} } \right)C_{33} - 2C_{13}^{2} } \right\}\left( {C_{55} + C_{66} } \right)} \right\}}}$$

5 $$B_{H} = \frac{{\left( {B_{V} + B_{R} } \right)}}{2}$$

6 $$G_{H} = \frac{{\left( {G_{V} + G_{R} } \right)}}{2}$$

The following equations can be used to derive Young's modulus (E) and Poisson's ratio from the bulk and shear moduli;

7 $$E = \frac{9BG}{{\left( {3B + G} \right)}}$$

8 $$\upsilon = \frac{{\left( {3B - 2G} \right)}}{{2\left( {3B + G} \right)}}$$

The Cauchy pressure for hexagonal structured materials can be approximated along two distinct orientations^44^.

9 $$P_{X}^{Cauchy} = C_{13} - C_{44}$$

10 $$P_{Z}^{Cauchy} = C_{12} - C_{66}$$

The elastic moduli are further used to estimate the hardness of both compounds by using the following expressions^45^:

11 $$H_{V} = 2\left( {K^{2} G} \right)^{0,585} - 3$$

For a material with a hexagonal structure, the shear anisotropy factor can be determined for three different crystallographic planes: (1,0,0), (0,1,0), and (0,0,1), using the following equations^46^:

12 $$A_{1} = \frac{{2\left( {C_{33} - 2C_{13} } \right) + C_{11} + C_{12} }}{{6C_{55} }}$$

13 $$A_{2} = 2C_{55} \left( {C_{11} - C_{12} } \right)^{ - 1}$$

14 $$A_{3} = \frac{{\left( {2(C_{33} - 2C_{13} ) + C_{11} + C_{12} } \right)\left( {C_{11} - C_{12} } \right)^{ - 1} }}{3}$$

The following equation can be used to obtain the linear compressibility coefficient (k_c_/k_a_)^47^:

15 $$\frac{{k_{c} }}{{k_{a} }} = \frac{{\left( {C_{11} + C_{12} - 2C_{13} } \right)}}{{\left( {C_{33} - C_{13} } \right)}}$$

## Results and discussions

This study represents the first comprehensive investigation into the equilibrium ground-state properties, as well as the thermodynamic and dynamical stability, of the quaternary Nb_2_ScAC_2_ (A = Al or Si) MAX phases. Furthermore, this study investigates the mechanical, thermal, and electronic characteristics of these compounds to enhance our understanding of their potential applications. Using the methods described above, the results for these MAX phases are presented in detail in this section, with each physical characteristic analyzed separately.

### Structural properties

**Figure 1:**
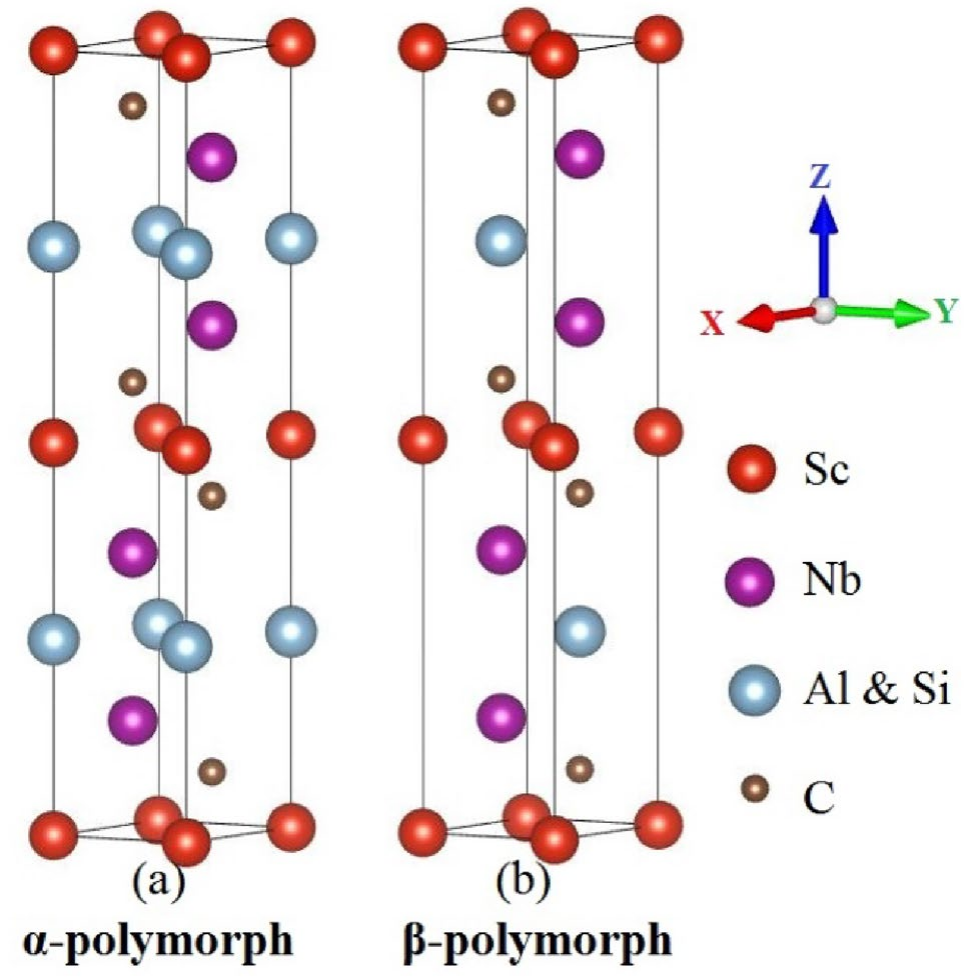
An illustration of the crystal structure of the MAX phases Nb_2_ScAC_2_ (A = Al & Si).

**Figure 2:**
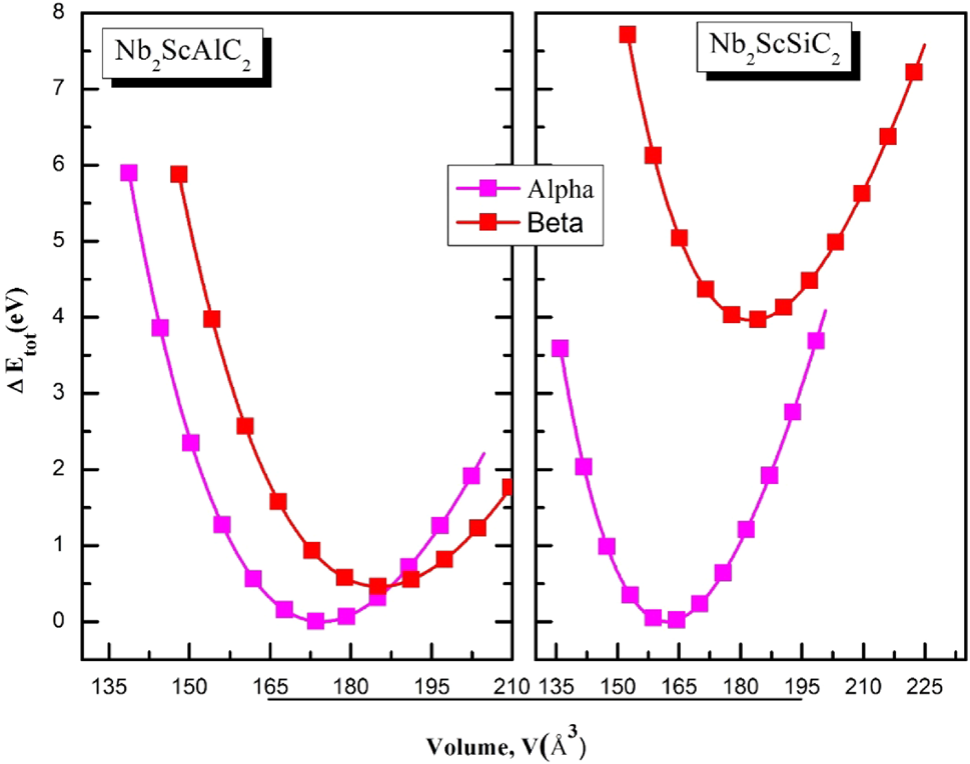
Changes in the energy with respect to volume for the quaternary Nb_2_ScAC_2_ compounds.

The hexagonal structure of space group P6_3_/mmc (No.194) is shared by both Nb_2_ScAlC_2_ and Nb_2_ScSiC_2_, which are MAX phase compounds. The unit cell of these MAX phase compounds is conventionally composed of 12 atoms, as depicted in Fig. 1. Table 1 presents the Wyckoff positions of Nb_2_ScAC_2_ (A = Al or Si), where ZM and ZC refer to the internal parameters of “Nb” and “C” atoms, respectively. In this study, the internal parameter c/a ratio and the (a, c) parameters were relaxed for each structure that was analyzed. The equilibrium cell volume was determined by optimizing the cell geometry using the conjugate gradient approach for total energy minimization. The energy vs. volume data for each structure was plotted and fitted using the Birch-Murnaghan EOS to determine the equilibrium ground state. The graphical representation of the overall energy with respect to volume for their two feasible configurations (α- and β-polymorph) is illustrated in Fig. 2. The lower total energy of the α-polymorph ensures that it is more stable for both Nb_2_ScAlC_2_ and Nb_2_ScSiC_2_ compounds. In order to assess the stability of our compounds and gain insights into their thermodynamic behavior, the calculation of formation energies is employed as the most reliable indicator. The formation energies of both compounds are determined using the following equation^48^:

16 $$E_{Form}^{{Nb_{2} ScAC_{2} }} = \frac{{E_{total}^{{Nb_{2} ScAC_{2} }} - aE_{tot}^{Mb} - bE_{tot}^{Sc} - cE_{tot}^{A} - dE_{tot}^{C} }}{a + b + c + d} ;\;\;A = Al, Si$$

**Table 1:** The Wyckoff positions of the polymorphs.

Atom	Site	Coordinates			
	Wyckoff	X	y	Z	Z_i_-range
α-polymorph					
M_I_(Sc)	4f.	0	0	0	**–**
M_II_(Nb)	2a	$\frac{1}{3}$	$\frac{2}{3}$	Z_M_	0.127277^1^/0.140169^2^
A(Al/Si)	2d	0	0	$\frac{1}{4}$	**–**
X(C)	4f.	$\frac{2}{3}$	$\frac{1}{3}$	Z_C_	0.07123^1^/0.07809^2^
β-polymorph					
M_I_(Sc)	4f.	0	0	0	**–**
M_II_(Nb)	2a	$\frac{1}{3}$	$\frac{2}{3}$	Z_M_	0.12023^1^/0.12302^2^
A(Al/Si)	2d	$\frac{1}{3}$	$\frac{2}{3}$	$\frac{1}{4}$	**–**
X(C)	4f.	$\frac{2}{3}$	$\frac{1}{3}$	Z_C_	0.06954^1^/0.07229^2^

^1^Nb_2_ScAlC_2_, ^2^Nb_2_ScSiC_2_.

where ${E}_{Form}^{{Nb}_{2}ScA{C}_{2}}$ is the formation energy of Nb_2_ScAC_2_ with (M = V and Nb), E_tot._ is the total energy per unit cell of the bulk compounds , and finally ‘a’, ‘b’, ‘c’ and ‘d’ indicates a number of Nb , Sc, (Al/Si) and C atoms in a unit cell of in the cell respectively. From Table 2 summarizes the lattice parameters, formation energies, and comparison studies for the two compounds, indicates that the Nb_2_ScAC_2_ compounds have negative formation energy, indicating their thermodynamic stability. It is noteworthy that Nb_2_ScSiC_2_ has particularly negative formation energy, indicating that it is more stable than Nb_2_ScAlC_2_. It should be noted that, as far as we know, no previous measurements or calculations have been conducted to determine the formation energy of Nb_2_ScAC_2_ compounds. Thus, our results can be regarded as a precise theoretical forecast.

**Table 2:** Computed structural parameters for both the compounds.

Compounds		Phase	a (Å)	c (Å)	c/a	B (GPa)	B’	Energy (eV)	E_form_ (eV/atom)
Nb_2_ScAlC_2_	Our work	Alpha	3.2369	19.2271	5.9398	175.9247	4.0254	− 474,799.4163	− 4.062
		Beta	3.1427	21.6585	6.8915	159.2836	4.0267	− 474,798.9553	− 4.023
Nb_2_ScSiC_2_		Alpha	3.2000	18.2819	5.7129	203.4167	4.0204	− 477,372.4966	− 3.164
		Beta	3.2232	20.3093	6.3009	172.4173	4.3757	− 477,368.54063	− 2.931
Mo_2_ScAlC_2_	^32^	Alpha	3.0524	19.0638	–	–	–	–	–

**Figure 3:**
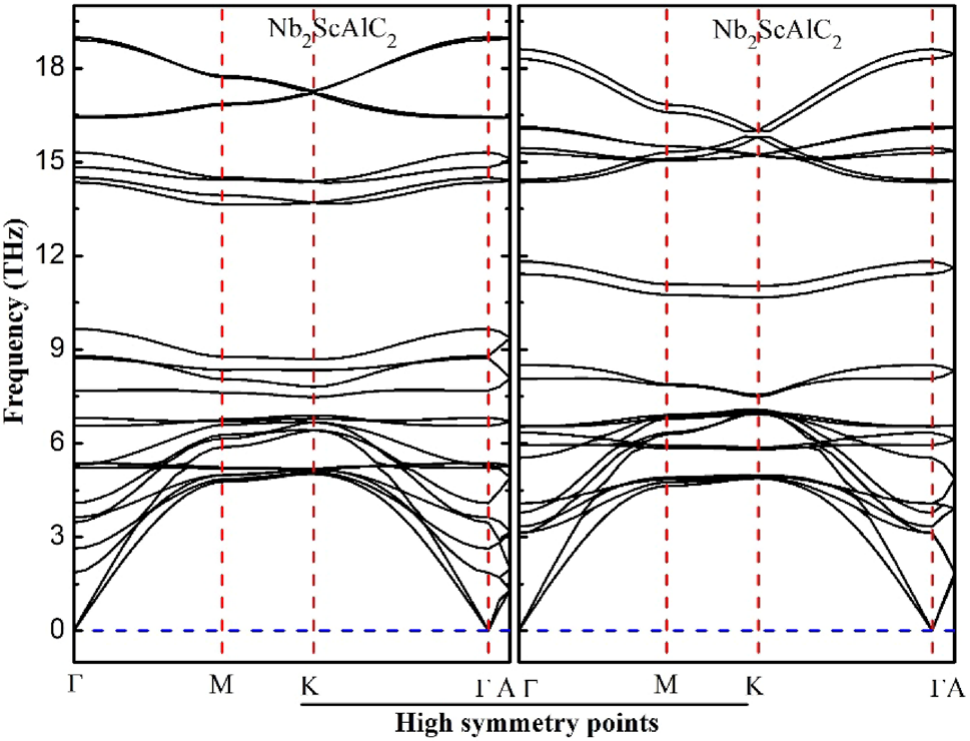
Calculated phonons dispersion for Nb_2_ScAC_2_ (A = Al, Si) compounds within the high symmetry directions of the initial Brillouin zone.

To verify the dynamic stability of the quaternary Nb_2_ScAC_2_ (A = Al or Si) MAX phases, we employed the finite displacement method, coded in the WIEN2K package, to calculate the phonon band structures of the compounds in the frequency interval of 0 THz to 30 THz. The hexagonal crystal structure of Nb_2_ScAC_2_ consists of 12 atoms per unit cell, while the phonon dispersion graph contains 36 branches consisting of 12 acoustic and 24 optic branched. The resulting phonon dispersion curves are displayed in Fig. 3. Notably, the dispersion relations for both substances exhibit only positive values, with the absence of negative values, which are an indication of imaginary phonon frequencies. This positive dispersion confirms the dynamical stability of the phonon modes in both compounds, providing further evidence for their overall thermodynamic stability. Moreover, the acoustic mode frequency is zero at the center zone point (Γ), robustly indicates that the Nb_2_ScAC_2_ is dynamically stable. So far, in our knowledge, phonon dispersion of both compounds has not been investigated theoretically or experimentally yet.

### Mechanical properties

The elastic constants can be employed to comprehend the mechanical characteristics of a material (C_ij_), which determine its response to external forces and provide insights into its mechanical properties. The mechanical stability and toughness of a material can be determined by these constants. To calculate elastic constants for a hexagonal crystal, the IR-elast package is used, which requires only five independent elastic constants. When a material is subjected to deformation within an elastic range, these constants can be used to link the mechanical response to the material's strength or weakness.

#### Elastic moduli and hardness

The elastic constants (C_ij_) can reveal information about the type of bonding between atoms, as they are the derivatives of the internal energy. The hexagonal structure of MAX phases results in only five independent C_ij_, namely C_11_, C_12_, C_13_, C_33_, and C_44_, subject to ensuring the subsequent requirements^49^:

17 $$\left\{ {\begin{array}{*{20}l} {C_{11} > \left| {C_{12} } \right| ,} \hfill & {2C_{13}^{2} < C_{33} \left( {C_{11} + C_{12} } \right)} \hfill \\ {C_{44} > 0 ,} \hfill & {C_{66} > 0} \hfill \\ \end{array} } \right.$$

Table 3 shows the elastic constants obtained from our calculations and compared with other theoretical studies. Our compounds satisfy the Born stability criteria, suggesting that they are mechanically stable against small elastic deformations. In order to evaluate the resilience of linear compression along the crystallographic a- and c-axes, the elastic constants C_11_ and C_33_ are calculated accordingly. Analysis of the elastic tensor indicates that for the compounds under study, C_11_ is higher than C_33_. This difference can be explained by the fact that along the a- and b-axes, the material exhibits higher resistance to compression along the a-axis compared to the c-axis. By analyzing the shear elastic constants C_12_ and C_13_, it was found that the maximum values were observed in the compounds under study in comparison to other compounds of the MAX phase, suggesting that both compounds are more effective in resisting shear deformation along the crystallographic c-direction than along the crystallographic a- and b-directions under certain stresses.

**Table 3:** Calculated elastic constants (C_ij_) (in **GPa**).

Compound		C_11_	C_33_	C_12_	C_13_	C_44_	C_66_
Nb_2_ScAlC_2_	Our work	338	315	88	105	146	125
Nb_2_ScSiC_2_		336	332	114	137	159	111
Mo_2_ScAlC_2_	^32^	293	290	109	117	134	92
Mo_2_TiAlC_2_	^33^	391	366	153	138	154	119

The stiffness of the material under investigation can be evaluated using the B, G, and E values obtained from the study. Table 4 presents the estimated values of B, E, and G. It is remarkable from Table 4 that Nb_2_ScAlC_2_ is less stiff than Nb_2_ScSiC_2_ which themselves are less stiff than the quaternary compound Mo_3_TiAlC_2_. However, compared to the same quaternary compound Mo_2_ScAlC_2_ (the Pugh’s ratio = 1.72) they are more brittle than this one. It should be noted that the Pugh's ductility index^50,51^, υ^52^ and the C_11_-C_44_ are the three conformist criteria used to assess the ductility and brittleness of the compounds. Nevertheless, the Pugh ratio carries greater significance as a criterion for determining the brittle or ductile nature of materials. The covalent or ionic bonding nature of a material can be determined based on the sign of the Cauchy pressure. Negative Cauchy pressure indicates a covalent bond, though positive Cauchy pressure indicates an ionic bond. A decisive value of 0.25 is assigned to Poisson's ratio^53,54^. Furthermore, an ionic bond is typically associated with a high value of Poisson's ratio, while a value below 0.25 is indicative of a covalent bond. Table 4 shows that both compounds have a Poisson's ratio below 0.25, indicating their covalent character, but the Nb_2_ScSiC_2_ compound will have a value close to 0.25 and this indicates the appearance of an ionic character, and it will appear clearly in the charge density, also through the Cauchy pressure for the two compounds.

**Table 4:** The following parameters were calculated for our compounds in two distinct orientations.

Compounds	Ref	B (GPa)	G (GPa)	B/G	E (GPa)	H_v_ (GPa)	ʋ	${P}_{X}^{Cauchy}$(GPa)	${P}_{Z}^{Cauchy}$(GPa)
Nb_2_ScAlC_2_	Our work	176.688	128.862	1.371	310.9	20.717	0.206	− 41.12	− 36.61
Nb_2_ScSiC_2_		197.992	124.413	1.591	308.6	16.518	0.240	− 22.30	2.923
Mo_2_ScAlC_2_	^32^	173	105	1.65	262	–	0.250	–	–
Mo_2_TiAlC_2_	^33^	222	133	–	331		0.251		

By using the Vicker’s formula, where K = G/B, we estimated the hardness of the compounds. It is apparent from the results that Nb_2_ScAlC_2_ has a higher hardness than Nb_2_ScSiC_2_. However, the values for both compounds fall within the range of hardness typically observed for MAX phases^55^.

#### Anisotropy factor

Evaluating the shear anisotropy factor of materials is crucial to gain insight into the extent of anisotropy in various physical occurrences. The k_c_/k_a_ can be determined from the equation mentioned earlier. A k_c_/k_a_ value of 1 signifies that the material is isotropic, while k_c_/k_a_ > 1 indicates that the compressibility along the a-axis is less than that of the c-axis. Similarly, k_c_/k_a_ < 1 indicates that the compressibility along the c-axis is less than that of the a-axis. Table 5 displays the anisotropy factors and compressibility coefficients. The value of the k_c_/k_a_ coefficient is higher than “1” for Nb_2_ScAlC_2_, whereas for Nb_2_ScSiC_2_ it is lower than 1 (0.90), indicating that the crystals exhibit higher compressibility along the c-axis than the a-axis for Nb_2_ScAlC_2_ and vice versa for Nb_2_ScSiC_2_. The distinction arises when replacing Al with Si, as Al forms a covalent bond while Si leads to the appearance of an ionic bond, creating a hybrid between covalent and ionic bonding. The results obtained for the electronic and mechanical properties are highly consistent with these findings.

**Table 5:** Shear anisotropic parameters were computed for three different shear planes (${{\varvec{A}}}_{1},{{\varvec{A}}}_{2} \; and \; {{\varvec{A}}}_{3}$) and ${{\varvec{K}}}_{{\varvec{c}}}/{{\varvec{K}}}_{{\varvec{a}}}$.

Compounds	Ref	${A}_{1}$	${A}_{2}$	${A}_{3}$	${K}_{c}/{K}_{a}$
Nb_2_ScAlC_2_	Our work	0.724	1.170	0.848	1.032
Nb_2_ScSiC_2_		0.593	1.431	0.850	0.906
Mo_2_ScAlC_2_	^32^	1.54			0.97
Mo_2_TiAlC_2_	^33^	1.30			1.10

### Thermal properties

The Debye temperature (θ_D_) is often used as a means of correlating physical properties such as thermal conductivity, thermal expansion, melting temperature, specific heat, and lattice vibrations of materials. This papers contain the final details for calculating these parameters and Table 6, presents the calculated ${\theta }_{D}$ values along with the sound wave velocities ${{\varvec{v}}}_{{\varvec{l}}}$, ${{\varvec{v}}}_{{\varvec{t}}}$, and ${{\varvec{v}}}_{{\varvec{m}}}$, as well as their corresponding literature values. The high Debye temperature of our compounds suggests that they may be a promising candidate for use as a thermal barrier coating (TBC) material when compared to other Mo_2_ScAlC_2_ and Nb_2_ScSnC_2_ compounds. Furthermore, the melting temperature of our compounds has also been calculated and is listed in Table 6. It is evident that our compounds exhibit a higher melting point than the compounds used for comparison, likely due to their associated elastic constants. Notably, our compounds have higher theoretical values than the comparison compound, the results suggest that these materials could potentially be useful in harsh environments.

**Table 6:** The properties that were computed include density, longitudinal and transverse elastic wave velocities, average elastic wave velocity (in units of g/cm^3^ and m/s), Debye temperatures (in Kelvin), melting temperature (in Kelvin), as well as minimum and lattice thermal conductivity at 300K (in units of W/m–K).

Compounds		ρ(g/cm^3^)	${v}_{l}$	${v}_{t}$	${v}_{m}$	*θ*_D_	${T}_{m}$	*k*_min_	k_ph_
Nb_2_ScAlC_2_	Our work	5.3637	8060.67	4901.51	5415.06	660.547	1842.642	1.256	46.03
Nb_2_ScSiC_2_		5.7943	7924.56	4633.73	5138.49	642.316	1863.012	1.251	34.49
Mo_2_ScAlC_2_	^32^	6.375	12,136	4058	4617	592.7	–	–	–

One of the most crucial parameters is the minimum thermal conductivity (κ_min_). For our investigation, we employed the Clark's model^56^:

18 $$\kappa_{\min } = k_{B} v_{m} \left( {\frac{{nN_{A} \rho }}{M}} \right)$$

Table 6 displays the calculated values of the minimum thermal conductivity (κ_min_) for the Nb_2_ScAlC_2_ and Nb_2_ScSiC_2_ solid solutions. It is noteworthy that both of our compounds exhibit the same minimum thermal conductivity values (0.603 W/m–K and 0.601 W/m–K, respectively). For thermal barrier coatings, a maximum limit of κ_min_ less than 1.25 W/m–K is considered appropriate. Consequently, we can conclude that both compounds may be utilized as materials for thermal barrier coating (TBC) applications^57^. The obtained result is highly favorable, as MAX phases possess an exceptionally symmetric crystal structure. The unique layered arrangement confers a remarkable degree of symmetry to the crystal lattice, making these materials extremely attractive for a wide array of applications, particularly as coating materials. On the other hand, we have employed Slack's model to calculate the lattice thermal conductivity (k_ph_)^58^.

Slack's model is an empirical formula, derived as follows:

19 $$k_{{{\text{ph}}}} = A\frac{{M_{{{\text{av}}}} \theta_{D}^{3} \delta }}{{\gamma^{2} n^{2/3} T}}$$

One of the variables in the equation is the dimensionless Grüneisen parameter, γ, which is obtained from the expression of Poisson's ratio:

20 $$\gamma = \frac{{3\left( {1 + \nu } \right)}}{{2\left( {2 - 3\nu } \right)}}$$

The factor A($\gamma )$ due to julian is calculated as:

21 $$A\left( \gamma \right) = \frac{{5.720 \times 10^{7} \times 0.849}}{{2 \times \left( {1 - \frac{0.514}{\gamma } + \frac{0.228}{{\gamma^{2} }}} \right)}}$$

**Figure 4:**
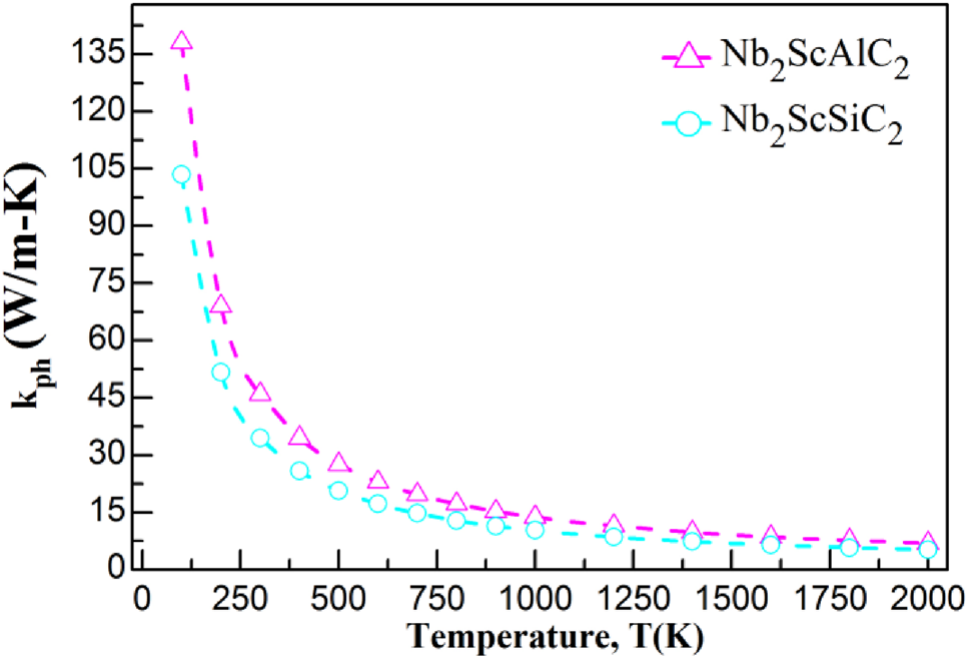
Temperature dependence of the lattice thermal conductivity of the Nb_2_ScAC_2_ (A = Al, Si) compounds.

Table 6 displays the calculated k_ph_ values at room temperature (300 K) for the materials under investigation. Figure 4 illustrates the dependence of k_ph_ on temperature within the range of (100-2000K). Lattice thermal conductivity is extremely susceptible to the Debye temperature. The credibility of the Slack model has been proven for MAX phases because their calculated lattice thermal conductivity matches fairly well with the experimental values. For example, the calculated (experimental) lattice thermal conductivity at 1300 K for Ta_4_AlC_3_ and Nb_4_AlC_3_ are 5 (6) W/m–K and 7 (7) W/m–K, respectively^9^. Notably, the replacement of Al with Si results in a disparity ratio of zero, indicating that the two compounds possess the same property. This feature also suggests that the material has potential as a thermal barrier coating (TBC)^59^.

To verify the suitability of studied compounds for TBC application their relevant properties are compared with those of promising TBC materials including MAX phases that are predicted as candidate materials for TBC application in the following Table 7.

**Table 7:** The properties that were computed include minimum thermal conductivity Debye temperatures, melting temperature, lattice thermal conductivity, as well as young modulus.

Compounds	Ref	*k*_min_ (W/m–K)	*θ*_D_ (K)	*T*_m_ (K)	*k*_ph_ (W/m–K)	*E* (GPa)
Nb_2_ScAlC_2_	Our work	1.256	660.5	1842.6	46.03	311.0
Nb_2_ScSiC_2_		1.251	642.3	1863.0	34.49	308.6
Y4Al2O9	^60^	1.12	564			191
Al_2_O_3_	^59^			2323		
Ti_3_ZnC_2_	^61^	1.229	617.9	1718.9	22.84	238
Ti_3_GaC_2_		1.399	702.1	1872.0	45.59	305
Ti_3_GeC_2_		1.420	707.0	1913.1	41.91	319
Ti_3_AlC_2_		1.550	780.7	1866.5	53.74	307
Ti_3_SiC_2_		1.631	806.4	1975.9	52.12	340
Ti_3_InC_2_		1.208	620.6	1782.2	36.31	279
Ti_3_SnC_2_		1.230	629.3	1808.8	36.24	294

### Electronic properties

**Figure 5:**
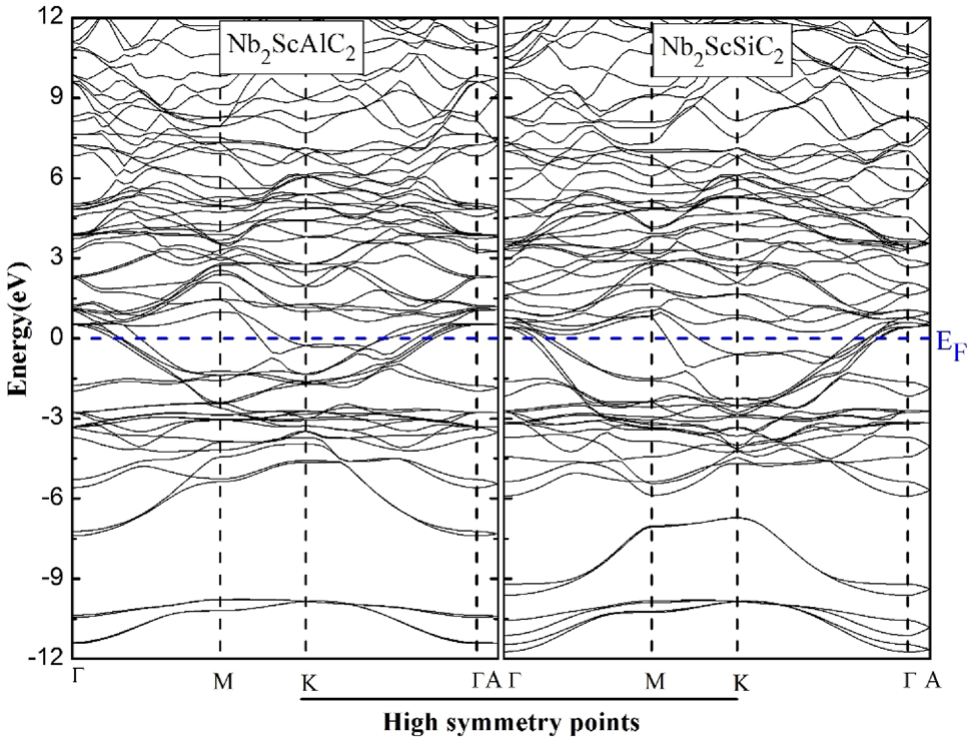
Band structure of the Nb_2_ScAC_2_ compounds within the high-symmetry directions of the initial Brillouin zone.

**Figure 6:**
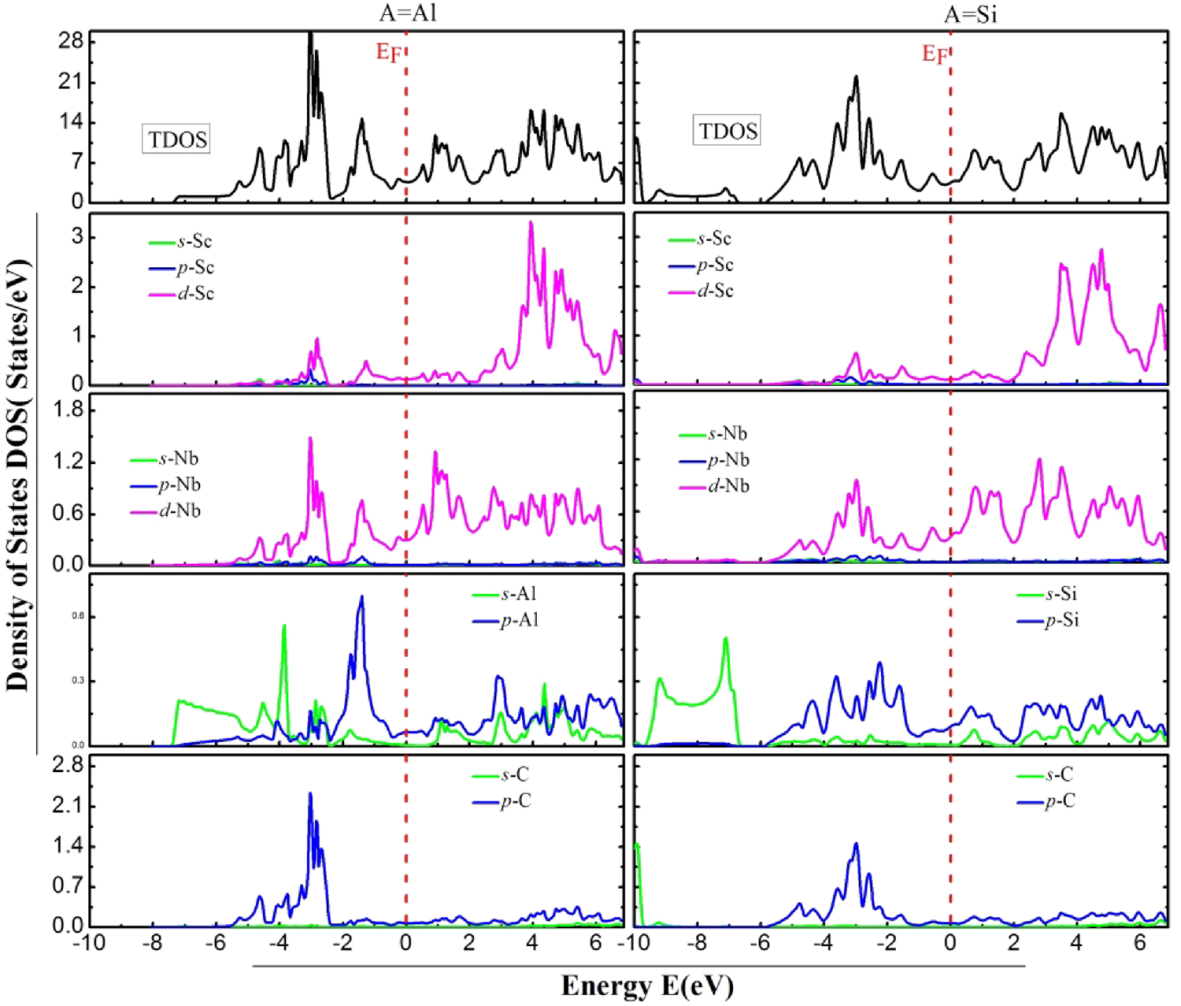
Electronic densities of states of Nb_2_ScAlC_2_ and Nb_2_ScSiC_2._

Studying the electronic properties of MAX phases is essential to gain a comprehensive understanding of them. These electronic properties, and the types of bonding that occur between their constituent elements along with the gap energies, are determined by their electronic band structure (EBS) and density of states (DOS). Hence, the analysis of EBS and TDOS is crucial to understand the electronic properties of these materials is depicted in Fig. 5, and Fig. 6, at first sight, we remark that the EBS, TDOS and partial density of states (PDOS) reveals the metallic character of the two compounds. It can be observed that multiple VBs intersect with the Fermi level and coincide with CBs. The (TDOS) and (PDOS) are calculated and plotted in Fig. 6. We can divide the spectrum into three regions founded on the analysis of the Partial Density of States (PDOS). For the Nb_2_ ScAlC_2_ we have the following:

From − 10 eV to –7eV is the VB dominated by the *s* states of silicon, the second region (− 7 eV to – 2.5 eV) a strong hybridization between the *d*(Nb) states and *p*(C) states and *d*(Sc) states. (-2.5 eV to E_F_ = 0 eV), the hybridization between the *p*(Al) states and the *d*(Nb) states. For the CB it is dominated by the *d*(Sc, Nb) states. For the Nb_2_ ScSiC_2_ compound, the lower VB is dominated by the *s*(Si) states. From (− 6 Ev to 0 eV) there is a hybridization between *p*(C) states *d*(Nb) states and a partial hybridization between the *p*(Si) states and *d*(Nb) states. Moreover, the CB is dominated by the *d*(Nb, Sc) states. This strong *pd* covalent bonding is mainly origin of the metallicity of our compounds. While the observed results in mechanical properties further substantiate this conclusion.

**Figure 7:**
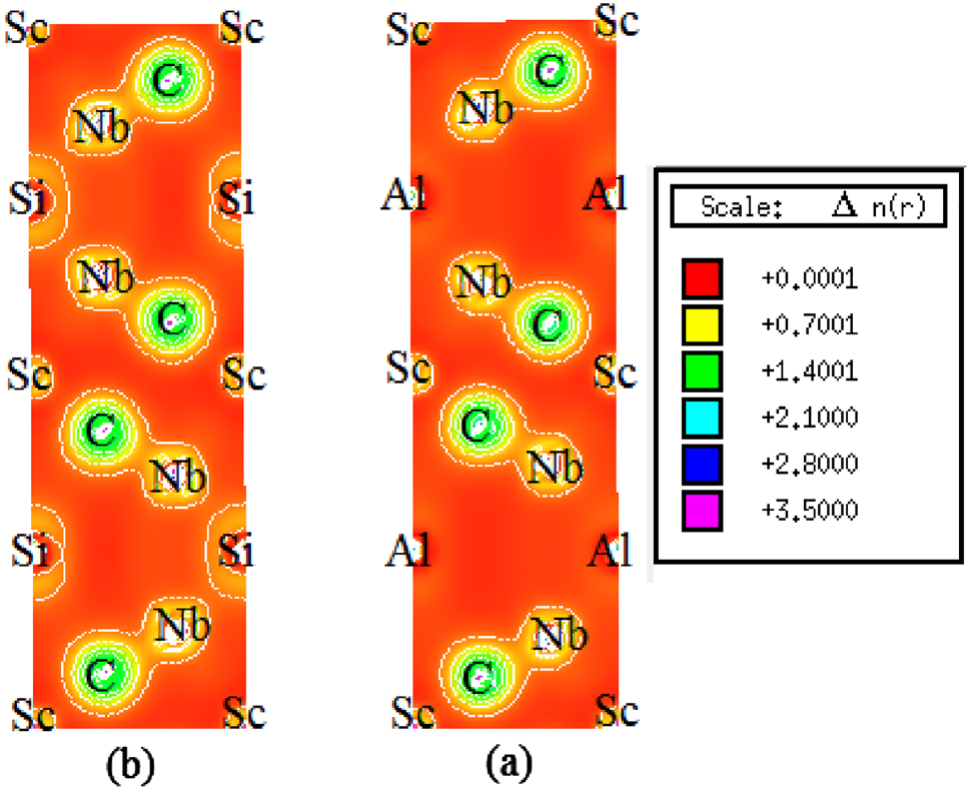
Charge density mapping image of (**a**) Nb_2_ScAlC_2_ and (**b**) Nb_2_ScSiC_2_.

The Figure demonstrates the calculated electron charge density mapping (in units of e/Å^3^) along the (110) crystallographic plane, aiming to provide a more detailed description of the chemical bonding nature in Nb_2_ScAC_2_. Figure 7 clearly reveals that the positions of C atoms and Nb atoms exhibit the highest accumulation of charge. This observation indicates the presence of strong covalent bonding between Nb and C atoms. The analysis of electronic properties indicates the presence of another covalent bonding between Nb and Si atoms, albeit with a comparatively weaker strength. This finding aligns with the results obtained from elastic constants, hardness measurements, and the density of states (DOS) analysis. It is worth highlighting that in both compounds, the covalent bonding between Nb and C is significantly stronger than that between Nb and Al for Nb_2_ScAlC_2_ but for Nb_2_ScSiC_2_ the covalent bonding between Nb and C is considered to be similar to the covalent bonding between Nb and Si. This observation is commonly observed in MAX phases, where the stacking of "hard" M-X bonds and "soft" M-A bonds along the c-direction contributes to this difference^4^. This finding is consistent with the correlation observed between the shear modulus, bulk modulus, and hardness.

### Optical properties

**Figure 8:**
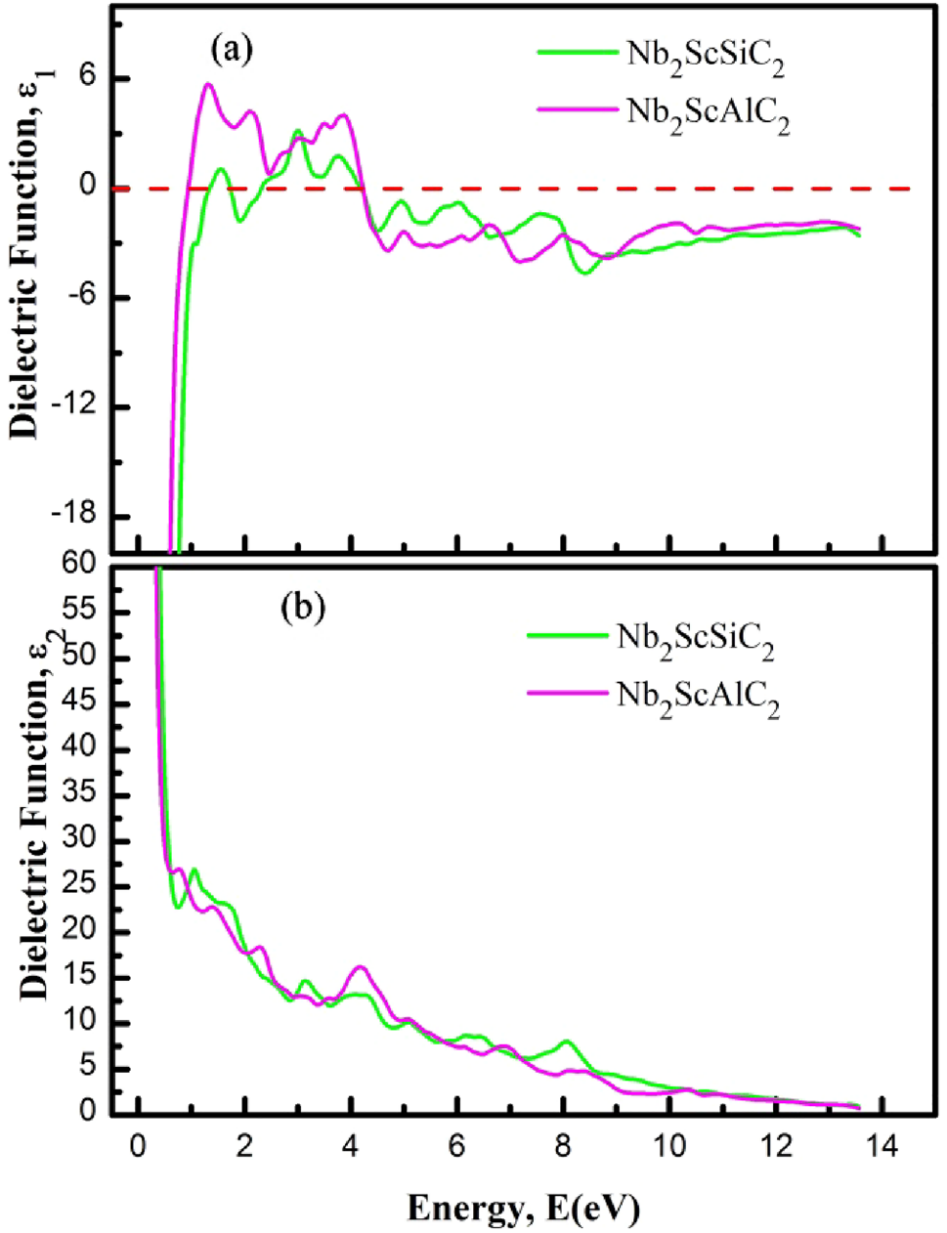
The (**a**) real of dielectric function (ε_1_), and (**b**) imaginary part of dielectric function (ε_2_) of Nb_2_ScAC_2_ (A = Al, Si) compounds.

Optical properties of materials have gained significant importance in the current age of information technology^62,63^. They are also important from the point of view of fundamental physics. The optical properties including dielectric constant, reflectivity and refractive index are calculated for the new layered semiconductor Nb_2_ScAC_2_ and their description with implications are given below:

The Fig. 8 shows the real and imaginary parts of the dielectric functions. The real part of the dielectric function ε_1_(ω) calculated for photon energy of 15 eV is shown in Fig. 8a. It can be said that the solid solutions exhibit highly anisotropic nature at the low energy region from 0 to ~ 4 eV. It is seen that the real part ε_1_(ω) goes through zero in the low energy range, indicating the metallic nature for all compositions of both compounds. The imaginary part of the dielectric function ε_2_(ω) represents the optical absorption of a material and is linked with the real part of the optical conductivity. The calculated ε_2_(ω) for Nb_2_ScAC_2_ is shown in Fig. 8b, For both polarization directions it approaches zero from above, indicating the metallic nature of all compositions. At the IR and visible light regions, the spectral features are different for the two polarization directions, highlighting the anisotropic nature in optical properties. At the high energy region the spectra for both polarizations are almost identical for all compositions. The electronic structure of a material is mostly accountable for optical spectra. For this reason, the origin of the peaks in the spectra can be explained from the DOS plot of the relevant material.

**Figure 9:**
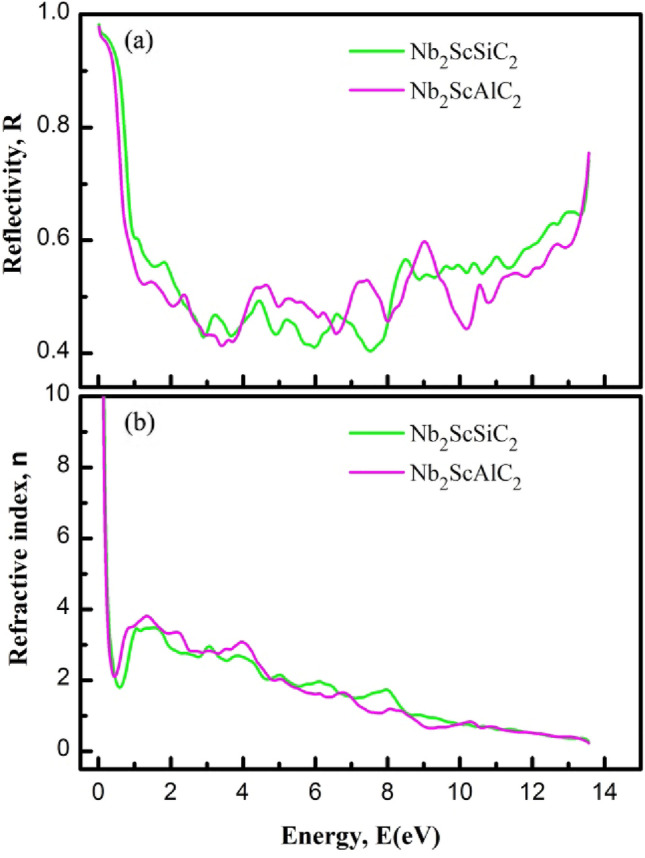
The (**a**) refractive index (n), and (**b**) reflectivity (R) of Nb_2_ScAC_2_ (A = Al, Si) compounds.

One of the significant applications of MAX phase materials is their use as coating materials to mitigate solar heating. The evaluation of this capability can be done by studying the reflectivity of the target materials. In our study, we have calculated the reflectivity spectrum of Nb_2_ScAC_2_ (A = Al, Si) compounds, illustrated in Fig. 9a. Previous research conducted by M. A. Ali et al. and Li et al.^64,65^ predicted that MAX phase materials with a reflectivity greater than 44% would effectively reduce solar heating. Based on the obtained reflectivity spectrum, it can be concluded that Nb_2_ScAC_2_ (A = Al, Si) compounds meet the criterion with a reflectivity greater than 44%. As a result, these materials have the potential to be utilized as effective coating materials for reducing solar heating.

The exact knowledge of the refractive index *n*(*ω*) of a material serves as a guide for perfect design of electronic devices. The refractive index is plotted in Fig. 9b. The static values of *n*(*0*) for Nb_2_ScAC_2_ (A = Al, Si) compounds are 5.91, and 6.64, respectively. These values represent the refractive index, indicating the phase velocity of incident radiation, and are specific to the mentioned compounds.

### Thermodynamic properties

**Figure 10:**
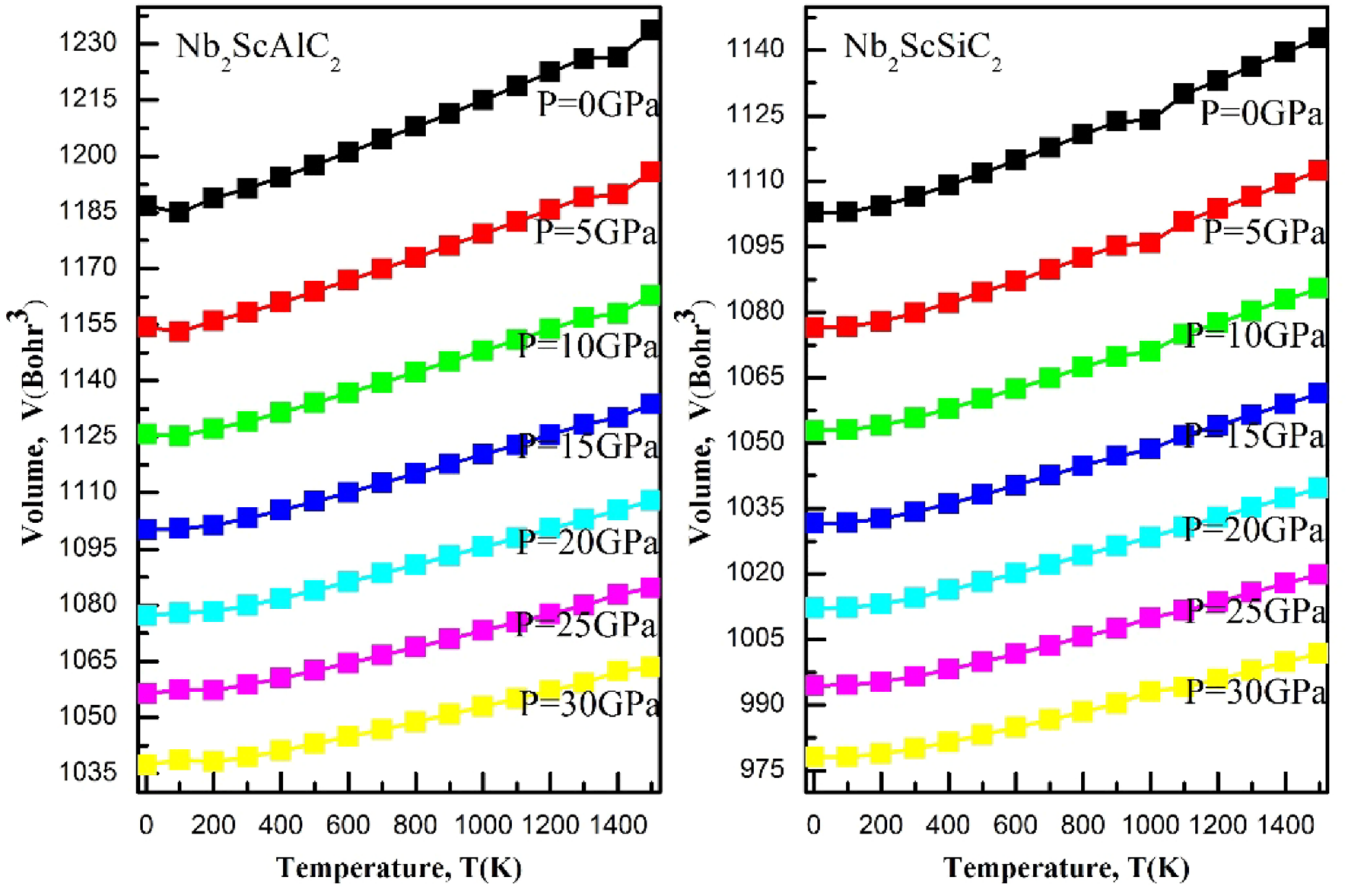
Volume as a function of the temperature at different pressures for the Nb_2_ScAC_2_ (A = Al, Si) compounds.

**Figure 11:**
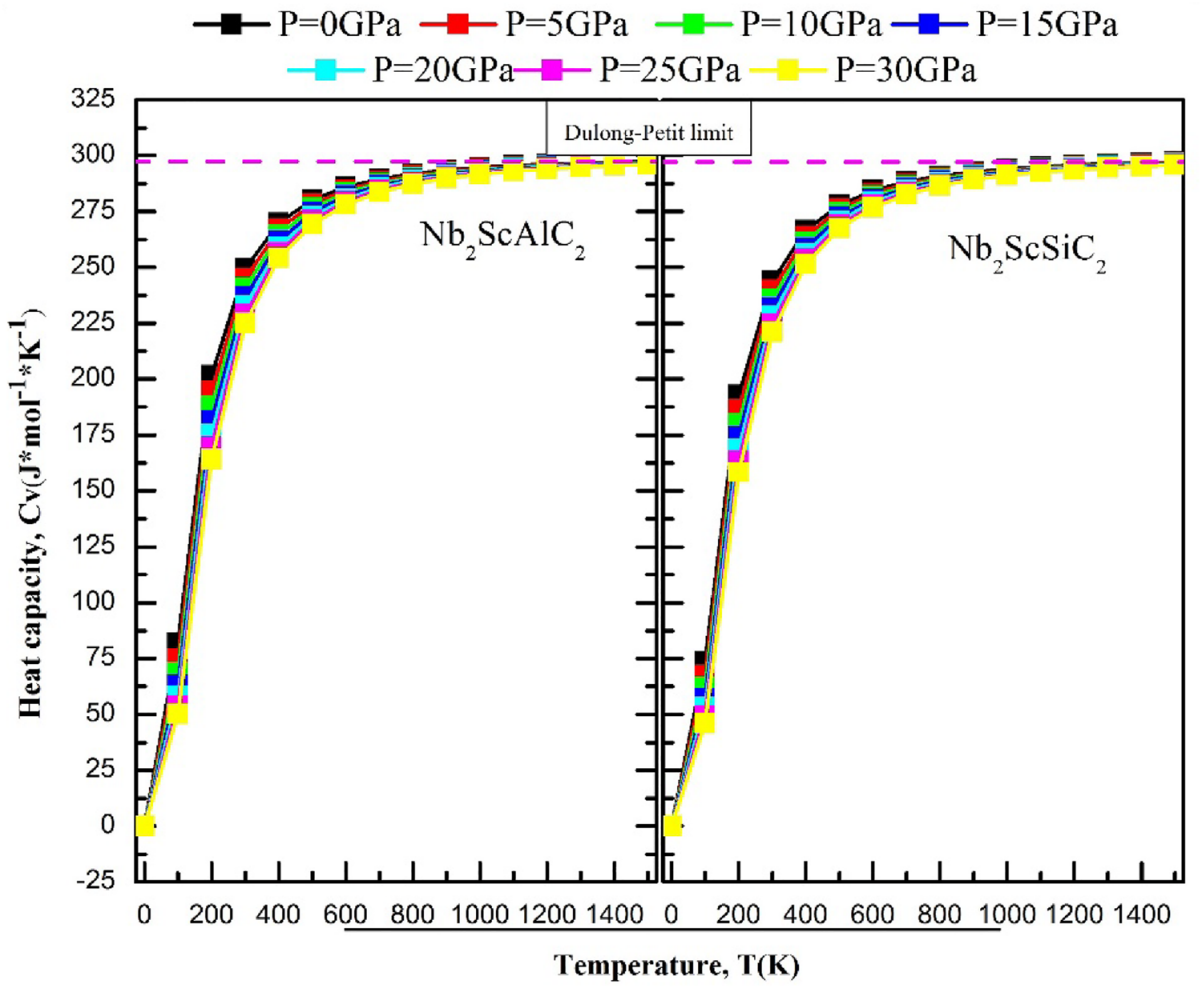
The graph shows the variation of heat capacity (C_V_) with temperature at different pressures for the Nb_2_ScAC_2_ (A = Al, Si) compounds.

**Figure 12:**
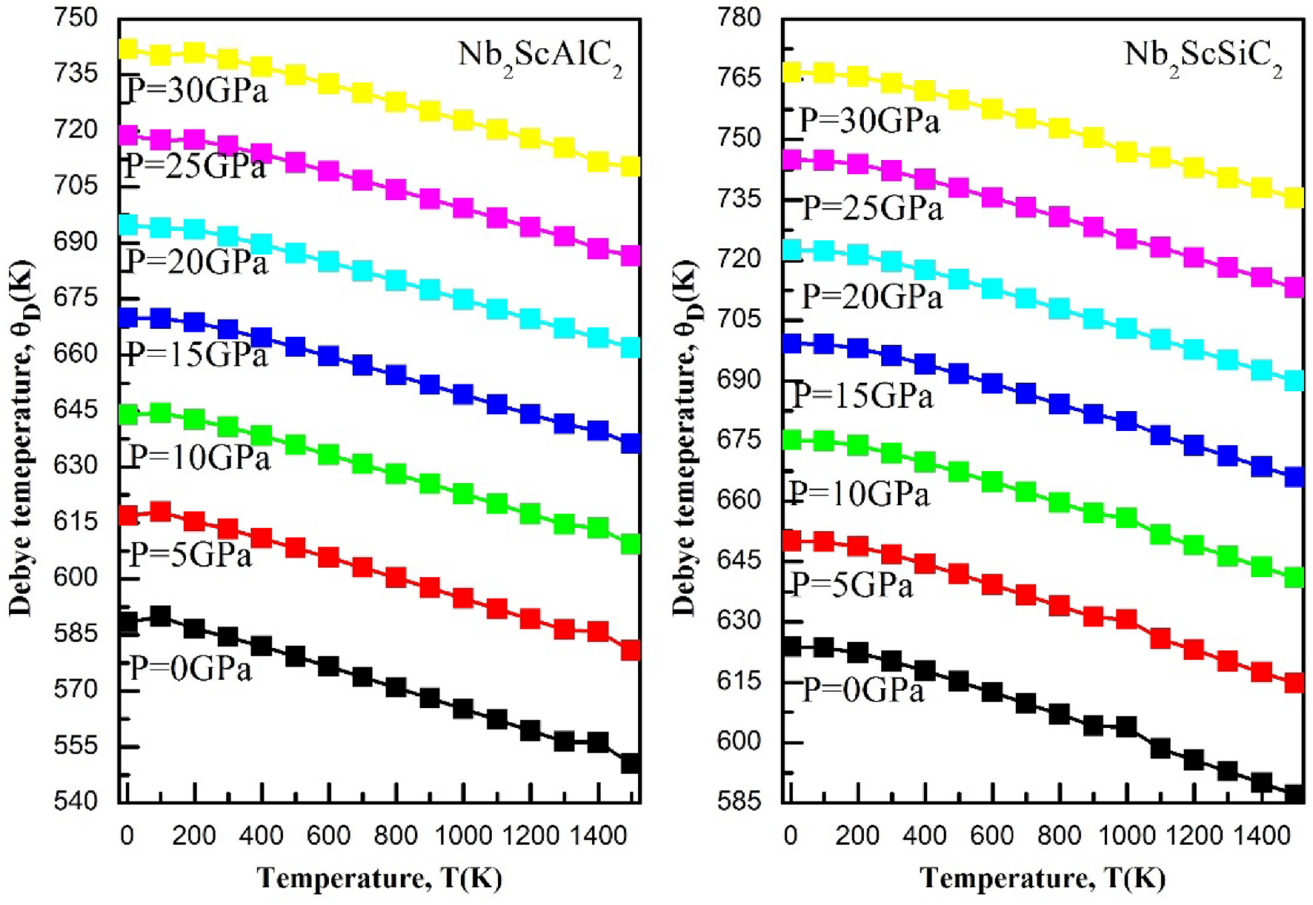
The variation of the Debye temperature (ƟD) with temperature at different pressures for the Nb_2_ScAC_2_ (A = Al, Si) compounds.

**Figure 13:**
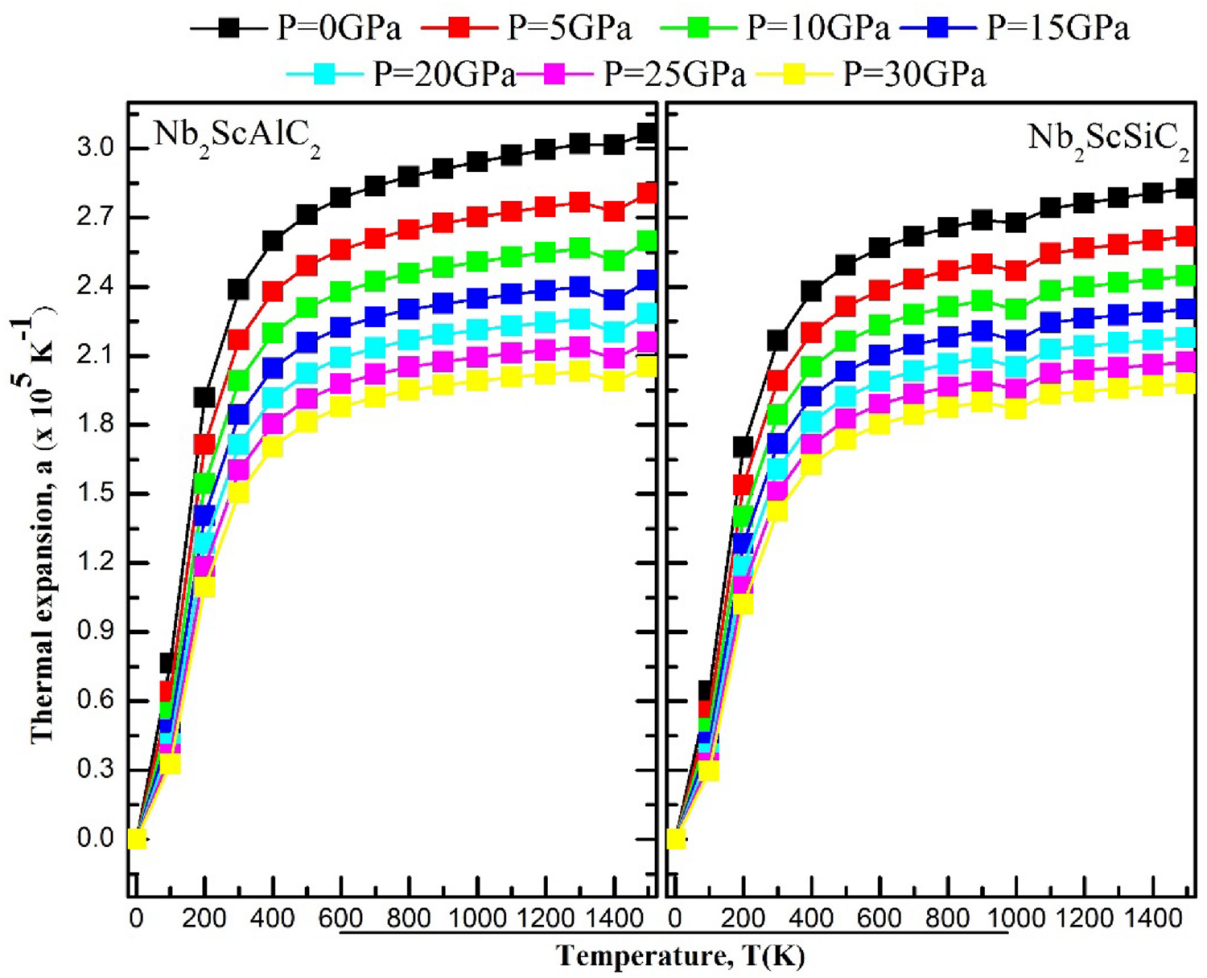
The thermal expansion coefficient as a function of temperature at various pressures for the Nb_2_ScAC_2_ (A = Al, Si) compounds.

A range of temperatures from 0 to 1500 K and a range of pressures from 0 to 30 GPa are explored to study the thermodynamic properties. The quasi-harmonic Debye model was employed in conjunction with the Gibbs package. The Debye model involves connecting a solid's elastic properties to its acoustic vibrations located at the center of the first Brillouin zone. Figure 10 illustrates the changes in volume for both compounds with respect to temperature and pressure. It is evident that, at a given temperature, the volume experiences a significant reduction with increasing pressure. On the other hand, as the temperature increases, the volume also increases. Therefore, the aforementioned findings strongly establish that alterations in temperature and pressure substantially impact the volume of the α-polymorph of both compounds. Figure 11 illustrates the changes in heat capacity at a constant volume with varying temperature at different pressures. For both compounds, the heat capacity at a constant volume approaches the Dulong-Petit classical limit at high temperatures. They exhibit a similar behavior to that of the ternary compounds V_3_SnC_2_ and the quaternary compound Mn_2_VSnC_2_. Within the temperature range of 0 K to 300° K, the heat capacity at constant volume c_v_, exhibits a rapid increase proportional to T^3^ and eventually attains a constant value at higher temperatures, adhering to the classical Dulong-Petit asymptotic limit. Figure 12 shows the Debye temperature, Ɵ_D_, as a function of temperature and pressure, the Debye temperature serves as an indicator of the strength of bonding in crystals. As temperature rises, atomic vibrations weaken the interatomic bonds, causing a decrease in Ɵ_D_. It can be observed that, at a given pressure, Ɵ_D_ decreases as the temperature increases. As the pressure increases up to 30 GPa, the Debye temperature experiences a significant increase. The thermal expansion coefficient α has been studied with varying temperature and pressure, their effects are shown in Fig. 13. Below 200K, the coefficients augment almost linearly. Exceeding 300K, its intensity reduces to 70% as the pressure increases similarly as the Mn_2_VSnC_2_ compound.

## Conclusions

A new MAX phase compounds based on Niobium has been predicted by using ab-initio calculations and study of their stability for their future use in smart coating. The Nb_2_ScAC_2_ (A = Al, Si) compounds, were stable in the α-polymorph structure. We calculated their formation energies which are negative ensuring the thermodynamic stability of the two compounds. The electronic band structure of the two compounds shows their metallic behavior. From the point of view of the elastic constants of the structure, the compounds are mechanically stable against elastic deformations. Thermodynamic analysis indicated that the effect of temperature escort the effect of the pressure on the Debye temperature whereas, the coefficient expansion exhibits the opposite effect of both temperature and pressure. The results revealed that these compounds are promising for smart coating and in harsh environment due to their high melting and Debye temperatures compared to quaternary compound like Mo_2_ScAlC_2_. Finally, we hope that this report will inspire experimentalists to synthesize these compounds in order to use them in future applications.

## Data Availability

Data can be provided on request made to the first author, Ahmed Azzouz-Rached.
